# Living in a bottle: Bacteria from sediment‐associated Mediterranean waste and potential growth on polyethylene terephthalate

**DOI:** 10.1002/mbo3.1259

**Published:** 2022-01-31

**Authors:** Àngela Vidal‐Verdú, Adriel Latorre‐Pérez, Esther Molina‐Menor, Joaquin Baixeras, Juli Peretó, Manuel Porcar

**Affiliations:** ^1^ Institute for Integrative Systems Biology (I2SysBio) University of Valencia‐CSIC Paterna Spain; ^2^ Darwin Bioprospecting Excellence S.L. Paterna Spain; ^3^ Cavanilles Institute of Biodiversity and Evolutionary Biology University of Valencia Paterna Spain; ^4^ Department of Biochemistry and Molecular Biology University of Valencia Burjassot Spain

**Keywords:** bioprospecting, bioremediation, marine sediments, marine waste, plastic‐degrading microorganisms, polyethylene terephthalate

## Abstract

Ocean pollution is a worldwide environmental challenge that could be partially tackled through microbial applications. To shed light on the diversity and applications of the bacterial communities that inhabit the sediments trapped in artificial containers, we analyzed residues (polyethylene terephthalate [PET] bottles and aluminum cans) collected from the Mediterranean Sea by scanning electron microscopy and next generation sequencing. Moreover, we set a collection of culturable bacteria from the plastisphere that were screened for their ability to use PET as a carbon source. Our results reveal that *Proteobacteria* are the predominant phylum in all the samples and that *Rhodobacteraceae, Woeseia, Actinomarinales*, or *Vibrio* are also abundant in these residues. Moreover, we identified marine isolates with enhanced growth in the presence of PET: *Aquimarina intermedia, Citricoccus* spp., and *Micrococcus* spp. Our results suggest that the marine environment is a source of biotechnologically promising bacterial isolates that may use PET or PET additives as carbon sources.

## Graphical abstract

Inner sediments from marine waste were analyzed using scanning electron microscopy, next generation sequencing, and culturomics. *Proteobacteria, Bacteroidota*, and *Actinobacteria* were predominant in all the samples. Enhanced growth on plastic by several microbial species was observed.

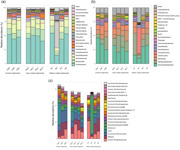

## 1 INTRODUCTION

Plastic production and, subsequently, plastic waste have increased exponentially through the last decades (Worm et al., 2017). The poor management of these residues, and their resistance to natural degradation (in some cases it comprises from hundreds to thousands of years) (Barnes et al., 2009), has resulted in a major, worldwide problem of plastic accumulation in all ecosystems on Earth. Even though the amount of recycled plastic has doubled from 2006 to 2018, the amount of post‐consumer waste plastic that is sent to landfills in Europe was still 25% in 2018 (PlasticsEurope, 2020).

Plastic residues in landfills are exposed to wind and water flows, which transport them into rivers and streams and, ultimately, into the oceans (Lebreton et al., 2017). Moreover, other direct sources such as beach littering, aquaculture, or fishing are also responsible for the accumulation of plastic in marine environments (GESAMP, 2016). Due to the generally low temperature and limited UV exposure in marine conditions, plastic degradation is considered to take longer in the sea (Gewert et al., 2015; Napper & Thompson, 2019). Plastic waste tends to fragment and spread in small particles (<5 mm) commonly known as microplastics (Arthur et al., 2009), which are easily ingested by marine wildlife, entering this way the trophic chain, and finally being ingested by humans (Setälä et al., 2014). Several studies have revealed the presence of plastic particles in fish, crustaceans, and mollusks (Neves et al., 2015; Van Cauwenberghe et al., 2015; Watts et al., 2014), and even in dietary salt (Iñiguez et al., 2017). This may have an impact on human health because of its physical accumulation as well as the toxicity of the additives used in plastic industries and the organic pollutants that plastic can adsorb in the marine environment (Bouwmeester et al., 2015; Rochman et al., 2013; Teuten et al., 2009). Moreover, not only the entrance of these microplastics on the trophic chain but also the enrichment of potentially pathogenic multidrug‐resistant bacterial strains in the plastisphere is a major health problem to face (Wang et al., 2021).

However, the amount of plastic estimated to enter into marine ecosystems does not correlate with the accumulation found by sampling techniques (Eriksen et al., 2014; Jambeck et al., 2015). Although there could be biases in sampling specific areas, this fact could also indicate that either physical or chemical plastic degradation is taking place in these ecosystems and/or microbial biodegradation is involved (Auta et al., 2017; Gewert et al., 2015; Sole et al., 2017; Zrimec et al., 2021). In recent years, plastic debris has proved a niche for specific plastic‐associated microbial communities to flourish, generally known as the “plastisphere” (Agostini et al., 2021; Zettler et al., 2013). Microbial growth on the plastisphere usually takes place in the shape of a biofilm on the plastic surface (Lobelle & Cunliffe, 2011). Although meta‐analyses are suggesting that a significant enrichment of potentially plastic biodegrading microorganisms in the plastisphere is detected (Wright, Langille, et al., 2021), there are still contradictory reports on the specificity of the composition of the microbial plastisphere. Specifically, some studies have shown that nonbiodegradable plastics, such as polyethylene terephthalate (PET), are colonized by a general biofilm rather than plastic‐specific species (Oberbeckmann et al., 2016; Pinnell & Turner, 2019). Therefore, microbial biofilms attached to plastic surfaces in the marine environment seem to be composed of complex communities where some microorganisms, although not being the primary producers, may have evolved or adapted to degrade plastic polymers or plasticizers (Pinnell & Turner, 2019).

In the last decades, there has been a rapid rise in the use of PET to produce disposable packaging, such as single‐use plastic bottles. This has led to a dramatic increase in PET waste generation, which is now one of the most common plastics polluting marine environments (PlasticsEurope, 2020; Ritchie & Roser, 2018). PET is a polymer made from raw petroleum‐derived monomers, terephthalic acid, and ethylene glycol. Its high content in aromatic compounds makes it chemically inert and subsequently very robust against biodegradation (Sinha et al., 2010).

In this context, bioprospecting microbial species able to in situ biodegrade plastic has arisen as a potentially useful tool for tackling the plastic contamination problem in the oceans (Danso et al., 2018). The first bacterium that demonstrated an effective PET‐degrading activity due to the expression of a lipase (PETase) was *Ideonella sakaiensis*, isolated from the sediments of a plastic‐recycling industry, which can hydrolyze this polymeric compound (Yoshida et al., 2016). However, these enzymes capable of PET hydrolysis have also been detected in other bacterial and fungal isolates, such as *Thermobifida fusca, Streptomyces* spp. or *Fusarium solani*, among others (Carr et al., 2020), and have been mainly described as cutinases, lipases, and esterases which are carboxylic ester hydrolases (Kawai et al., 2020).

Here, we show a complete characterization of the microbial communities associated with marine residues from the Mediterranean Western coast with a dual culture‐dependent and ‐independent approach. We have studied the biofilm morphology on plastic and aluminum debris through scanning electron microscopy (SEM), characterized the microbial communities of their inner sediments by 16S and 18S ribosomal RNA (rRNA) genes sequencing, and established a microbial collection of mainly culturable bacteria and some yeasts, whose ability to grow on media supplemented with PET as sole carbon source has been characterized.

## 2 MATERIALS AND METHODS

### 2.1 Sampling

**Figure 1:**
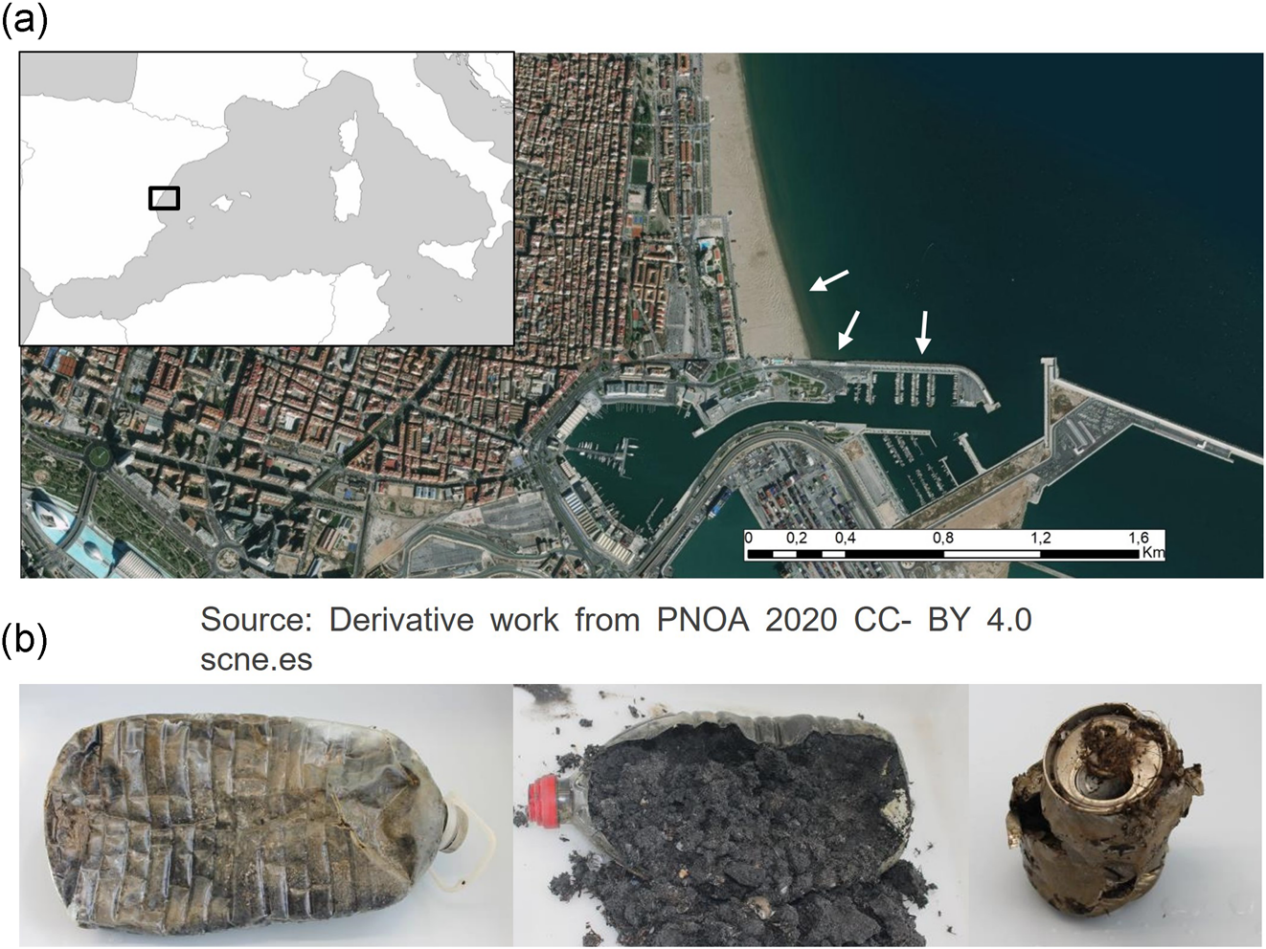
(a) Sampling location at the Mediterranean Western coast, Malva‐rosa beach, València (Spain). The specific sampling sites are pointed out with white arrows. (b) Examples of the samples collected, from left to right: PET plastic bottle P1; plastic bottle P2; aluminum can M10

Plastic residues and cans were collected from the Malva‐rosa beach (València, Spain; 39°27′48.3″N 0°19′07.6″W) in September 2017 (Figure 1). The sampling was carried out at 20 m from the coastline and 3 m in depth. Four PET plastic bottles (labeled as P1–4) and four metallic beverage cans (labeled as M10–13) were collected and transported to the laboratory into sterile plastic bags. All the residues were originally submerged or half‐buried in the marine sediments and they were thus partially filled with sand, mollusk shells, and marine plants (*Posidonia oceanica*) debris. Three samples of control seabed sediments (CS4–6) from the same area where plastic and aluminum residues were collected, which consisted of similar materials like sand, little stones, and shells, were also collected. Furthermore, some of the marine residues collected were still labeled with the expiration date of the product; therefore, an approximate age for these bottles or cans can be deduced: aluminum can M10 (expiration date 2003), aluminum can M12 and M13 (expiration date 2018), plastic bottle P1 (expiration date 2010).

Samples from the insides of each recipient (sediments) were collected under sterile conditions in the laboratory and stored at −20°C until required. To obtain samples from the plastic surface biofilms, recipients P1–4 were shortly rinsed with sterile water and then cut into small pieces which were shaken together with glass beads in phosphate‐buffered saline (PBS; pH 7.4; in g/L: 8.0 NaCl, 0.2 KCl, 1.42 Na_2_HPO_4_, 1.80 KH_2_PO_4_), at 500 rpm, for an hour. A total of 150 ml of the resulting suspension were collected and centrifuged at 4500 rpm for 15 min (sample P12) and stored at −20°C until required. Sample 12 was only analyzed in terms of culturable bacteria and it was not included in the high‐throughput 16S rRNA gene sequencing.

### 2.2 Isolation of microbial strains

Sediment samples from recipients P1–4, M10–13, biofilm sample P12, and control sediments CS4–6 were diluted in PBS at a final ratio of 1:4 (v:v). Serial dilutions were then prepared and four replicates of 50 µl aliquots were spread on commercial Marine Agar (MA) (Ref: 1059; Laboratorios Conda S.A.) and incubated at 18°C for 2 weeks. Two replicates were incubated under aerobic conditions and the other two replicates in anaerobic conditions by placing the dishes inside a hermetic container without oxygen (N_2_ atmosphere).

Individual colonies were picked according to morphological traits (color, shape, and size) and restreaked on fresh media until a pure culture was obtained. The strains were named after a code composed of a letter and a number associated with its origin (P1–4 and P12: plastic bottles; M10–13: aluminum cans; CS4–6: external sediments), followed by a unique number for each strain and a letter referring to the incubation conditions (X: aerobic conditions; A: anaerobic conditions). For example, P1.1X means the first colony isolated from bottle P1 that grew under aerobic conditions. The strains were stored in cryotubes with 20% glycerol at −80°C until used.

### 2.3 Molecular identification of isolates through 16S/18S rRNA gene sequencing

DNA extraction was carried out by using the protocol described by Latorre et al. (1986) and confirmed through electrophoresis in agarose gel (1.4% w/v). Strain identification was performed through 16S rRNA gene Sanger sequencing, by using the universal primers 8 F (5ʹ‐AGAGTTTGATCCTGGCTCAG‐3ʹ) and 1492 R (5ʹ‐CGGTTACCTTGTTACGACTT‐3ʹ). In the cases that the 16S rRNA gene amplification failed, 18S rRNA gene universal primers 86 F (5ʹ‐ACTGCGAATGGCTCATTAAATCAG‐3ʹ) and 1188 R (5ʹ‐AGTCAAATTAAGCCGCAG‐3ʹ) were used to verify whether the strains were eukaryotic. Amplicons were precipitated overnight in isopropanol 1:1 (v:v) and potassium acetate 3 M, pH 5, 1:10 (v:v) at −20°C. After centrifuging at 12,000 rpm for 10 min, DNA pellets were washed in 70% ethanol and resuspended in the required amount of sterile Milli‐Q water. BigDye® Terminator v3.1 Cycle Sequencing Kit (Applied Biosystems) was used for amplicon tagging for Sanger sequencing, which was performed in the Sequencing Service (SCSIE) of the University of València (Spain). The Sequences were manually edited with Trev (Staden Package, 2002) to eliminate low‐quality base calls and compared by EzBioCloud 16S online tool (https://www.ezbiocloud.net/). The 16S rRNA genes of some interesting isolates holding an identity lower than 98.7% with the closest type strain were also sequenced with primers 341 R (5ʹ‐ CTGCTGCCTCCCGTAGG‐3ʹ) and 1055 F (5′‐ATGGCTGTCGTCAGCT‐3′) and complete 16S rRNA gene sequences were assembled with the MEGA10 tool and compared again by EzBioCloud 16S online tool.

### 2.4 Scanning electron microscopy

Plastic and aluminum samples were briefly washed with sterile distilled water and then pieces of ca. 0.25 cm^2^ were cut and fixed in Karnovsky's fixative (Karnovsky, 1965). The fixation solution was changed after five hours and samples were stored in this solution at 4°C until required. For SEM, the pieces were washed in phosphate buffer 0.1 M, pH 7.4 (PB, in g/L: 3.1 NaH_2_PO_4_·H_2_O, 10.9 Na_2_HPO_4_) to remove the fixative and progressively dehydrated in increasing ethanol concentrations. Samples were placed inside microporous specimen capsules (30 μm pore size) immersed in absolute ethanol, followed by critical point drying in an Autosamdri 814. The fragments were then arranged on SEM aluminum stubs using carbon tape and coated with Au/Pd sputtered in argon gas. The observation was carried out in a Scanning Electron Microscope Hitachi S‐4800 at the electron microscopy service of the University of València (SCSIE).

### 2.5 DNA purification and high‐throughput 16S rRNA gene sequencing

Internal sediments from the marine residues collected were subjected to DNA extraction. In particular, 1 g of sediments of each sample (Plastic bottles P1, P2, P3, and P4; Aluminum cans M10–M13; Control sediments CS4–CS6) were taken from 2 cm in depth from the inner sediments of each bottle/can. No replicates were performed. Metagenomic DNA extraction was carried out by using the Power Soil® DNA Isolation Kit (12888‐100; MoBio Laboratories Inc.) according to the manufacturer's instructions, but incubating at 65°C (10 min) after the addition of solution C1, and resuspending the extracted DNA in 25 µl of Milli‐Q water. The resulting DNA was quantified using the QUBIT dsDNA HS‐high sensitivity kit (Invitrogen). Then, primers 341 F (5′‐CCTAYGGGRBGCASCAG‐3′) and 806 R (5′‐GGACTACNNGGGTATCTAAT‐3′) were used to amplify the V3–V4 region of the 16S rRNA gene. All polymerase chain reactions (PCRs) were carried out with Phusion® High‐Fidelity PCR Master Mix (New England Biolabs). PCR products were mixed at equal density ratios. The pool was then purified with Qiagen Gel Extraction Kit (Qiagen). Sequencing libraries were generated with NEBNext® Ultra™ DNA Library Prep Kit for Illumina and quantified via Qubit and qPCR. Finally, the NovaSeq 6000 Sequencing System (2 × 250 bp) was employed for sequencing the samples. All the library preparation and sequencing steps were carried out by Novogene.

### 2.6 Bioinformatic analysis

Raw Illumina sequences were analyzed using Qiime2 (v. 2020.8) (Bolyen et al., 2019). Briefly, the quality of the reads was assessed with the Demux plugin, and the sequences were subsequently corrected, trimmed, and clustered into amplicon sequence variants (ASVs) via Dada2 (Callahan et al., 2016). The taxonomy of each sequence variant was assigned employing the classify‐Sklearn module from the feature‐classifier plugin (Bokulich et al., 2018). SILVA (v. 138) was used as a reference for the 16S rRNA gene assignment (Quast et al., 2013). The phyloseq R package (McMurdie & Holmes, 2013) was used for analyzing and visualizing the data. All the α‐diversity tests were carried out using ASVs and rarefying to the lowest library size (128,327 sequences). Principal coordinate analysis (PCoA) plots were created using Bray–Curtis as a dissimilarity measure. Finally, DESeq. 2 (Love et al., 2014) was used for differential abundance analyses).

### 2.7 Plastic degradation assay in solid medium

Plastic degradation was assessed through qualitative assays by comparing the growth of the bacterial strains on minimal marine medium (MMA), enriched marine medium (MME), and marine medium supplemented with plastic (MMP). MMA consisted of water from the Mediterranean Sea and 15 g/L agar, whereas MME consisted of seawater and, in g/L, 1.0 yeast extract, 5.0 bacteriological peptone, and 15 agar. MMP was prepared by using seawater, supplemented with 9.3 g/L of ground PET of approximately 0.5 mm in size, from a commercial PET water bottle (brand Cortes) and 15 g/L of agar, which was then sterilized at 121°C for 30 min. The PET bottle was ground in a coffee grinder for 5 min at maximum speed. As plastic particles tended to sediment on the bottom of the dishes, the media was stirred by using sterile spatulas before solidification.

Before the incubation with PET, bacterial isolates were grown on solid MMA for 4 days at room temperature. Cell suspensions with an Optical Density at 600 nm (OD_600_) of 1 were prepared in PBS and 4 µl of the suspensions were placed on Petri dishes containing MMA, MME, and MMP (in duplicate). The dishes were incubated for 16 days at 18°C. Isolates with a more vigorous growth (as determined by colony diameter and cell density) in MMP than in MMA were selected as potential plastic degrading bacteria and tested again in the same media conditions but using a 10‐fold dilution of the bacterial suspensions (OD_600_ of 0.1).

### 2.8 Plastic degradation assay in liquid medium

Assay tubes were prepared with 3 ml of seawater and 0.400 ± 0.001 g of particles of PET from a new water bottle (brand Cortes), of 3 mm in size (cut by hand to obtain homogeneous size), and sterilized by autoclaving at 121°C for 30 min. Bacterial strains were grown on solid MA for 4 days at room temperature. Cell suspensions were prepared in PBS and adjusted to a final OD_600_ of 0.05. The assay was carried out in duplicate by incubating the tubes at 18°C under shaking (200 rpm) for 3 months. Control tubes consisted of sterile seawater inoculated with the microbial cultures, as well as seawater and plastic particles but without inoculated bacteria.

At the end of the incubation period, PET fragments were rinsed with sterile water and vortexed for 2 min in distilled water. The process was repeated three times and the washed plastic particles were dried at 65°C for 48 h. Finally, the remaining plastic particles were weighted in a precision balance. To finally compare the colony‐forming units (CFU) in each condition, the recovered supernatants of each tube were diluted in serial dilutions and 50 µl of each dilution was inoculated in duplicate into MA plates.

## 3 RESULTS

### 3.1 Residue types and samples

Plastic PET bottles and aluminum cans were collected to study their associated microbiota as described in Section 2. The bacterial communities present in the inside‐sediments, coming from PET bottles and aluminum cans, were compared with control, non‐artificial residues‐associated sediments from the same area. Interestingly, some of the marine residues collected were still labeled with the expiration date of the product; therefore, an approximate age for these bottles or cans can be deduced: aluminum can M10 (expiration date 2003), aluminum can M12 and M13 (expiration date 2018), plastic bottle P1 (expiration date 2010).

### 3.2 Scanning electron microscopy

**Figure 2:**
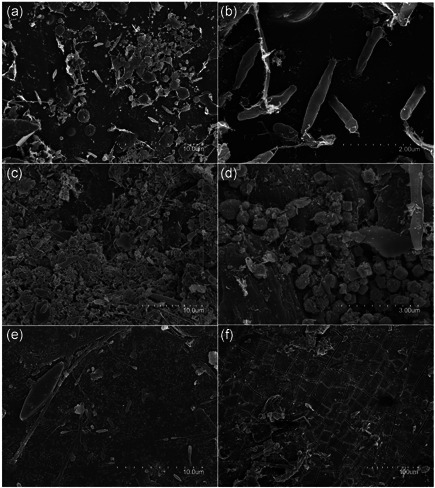
Scanning electron microscopy images of microorganisms on the surface of different marine residues. Scale bar (a) 10 µm, (b) 2 µm, (c) 10 µm, (d) 3 µm, (e) 10 µm, and (F) 100 µm. (a, b) Microbial community on the plastic surface of sample P1. Fusiform bacilli‐like microorganisms attached to the surface by fimbriae‐like adhesion structures. (c, d) Biofilm on the plastic surface of sample P2. Square‐like nonidentified shapes of less than 1 µm are predominant in this sample. (e, f) The surface of aluminum cans with scattered microbial cells

The SEM images of the surface of plastic and aluminum marine waste suggest a diverse microbial community attached to these surfaces (Figure 2). Different microbial morphologies could be differentiated in both cases, including rod‐ and coccus‐shaped cells as well as diatoms and filamentous microorganisms. In particular, spermatozoid‐shaped bacteria stood out in Figure 2c,e which may belong to prosthecate bacteria such as *Hyphomonadaceae*. Interestingly, several samples showed 2 µm fusiform bacilli firmly attached to the plastic surface, to which they were linked through polar fimbriae‐like structures (Figure 2a,b). In another plastic bottle, one of the most frequent morphologies was a square shape of around 0.6 µm in size which could not be attributed to a microorganism as it could instead correspond to mineral forms (Figure 2c,d). Finally, eukaryotic flagellated cells and diatoms were observed in the analyzed aluminum surfaces of cans (Figure 2e,f).

### 3.3 Taxonomy of the waste‐associated bacterial communities

**Figure 3:**
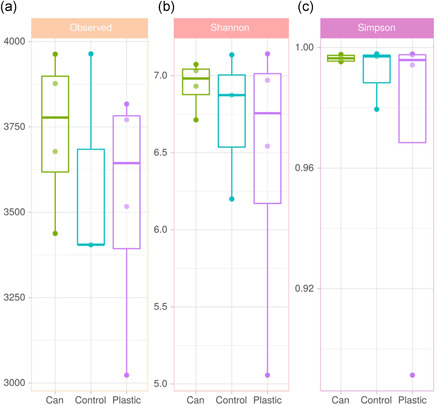
Representations of the values of alpha diversity indices in the (a) observed richness at the amplicon sequent variant (ASV) level (number of ASVs), (b) Shannon index of diversity, and (c) Simpson index of diversity. The 11 analyzed samples are represented: inside‐sediments of cans (green); polyethylene terephthalate inside‐sediments (purple); control‐sediments of the sea‐bed (blue)

**Figure A1:**
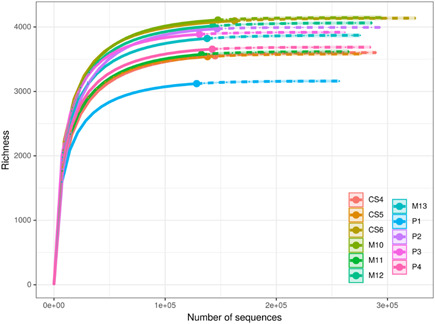
Rarefaction curves

The bacterial community of marine waste was studied by high‐throughput 16S rRNA gene sequencing yielding the composition of the taxa in the inside sediments of four PET bottles, inner sediments of four aluminum cans, as well as three samples of control marine sediments. The shape of rarefaction curves revealed that sequencing was deep enough to cover all the microbial diversity for all samples (Figure A1). Furthermore, based on the comparison of the richness value (number of different AVSs; Figure 3a) and the diversity (Shannon index; Figure 3b and Simpson index; Figure 3c), the alpha diversity was not significantly different among samples (*p* > 0.1; Mann–Whitney *U* test).

**Figure 4:**
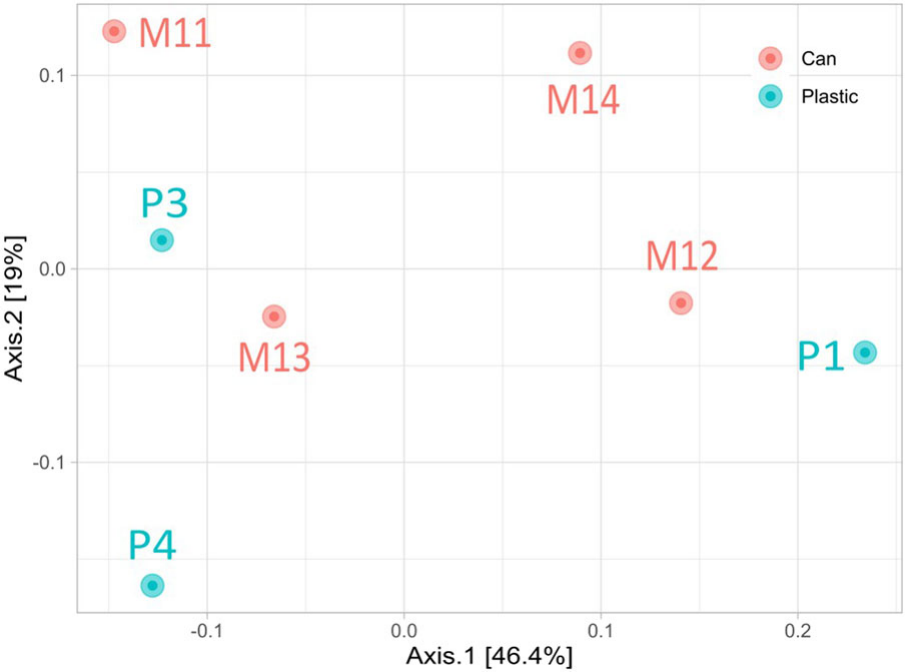
Principal coordinates analysis (PCoA) based on Bray–Curtis dissimilarities at the genus level in bacterial populations of both inside‐sediments of marine residues, plastic (blue), and aluminum cans (red). Sample P2 not included

**Figure A2:**
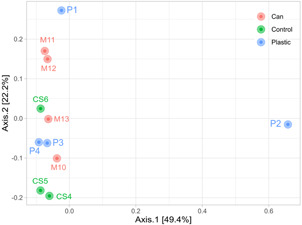
Principal coordinate analysis including all samples

A PCoA including samples P1, P3, P4, M10, M11, M12, and M13 revealed no significant difference between the composition of the bacterial communities of both (plastic and cans) inside waste sediments (*p* > 0.05; PERMANOVA) (Figure 4). Sample P2 was not included in Figure 4 due to its substantial difference in bacterial composition, which precluded its separation from the other samples in the PcoA and difficulted the interpretation of the figure (see Figure A2 for the complete analysis).

**Figure 5:**
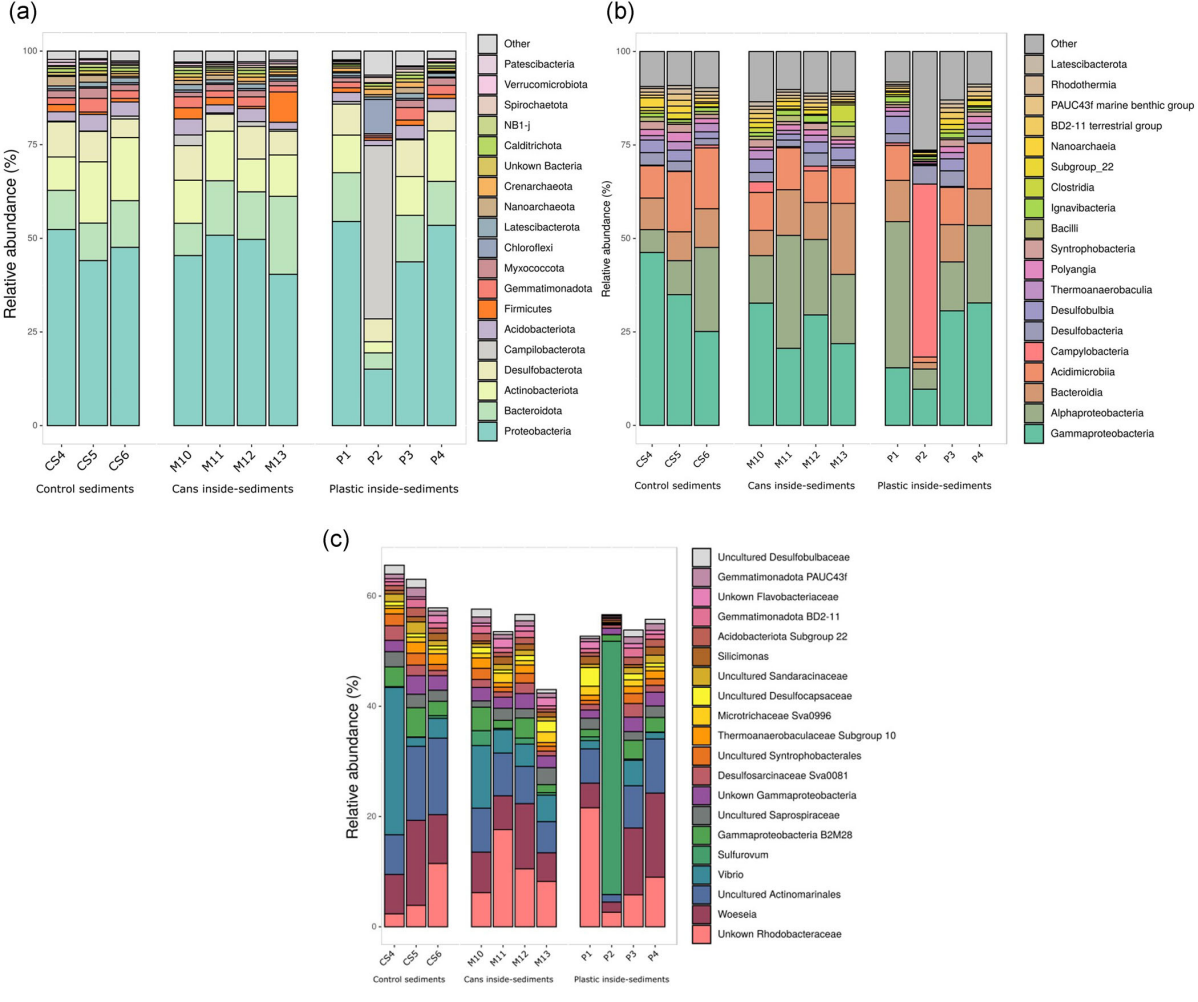
Barplots showing the taxonomic profiles at the phylum (a), class (b), and genus (c) level of the top 20 most abundant groups in terms of relative abundance of inside‐sediments from marine residues (plastic and aluminum cans) and control sediments by high‐throughput 16S ribosomal RNA gene sequencing

The representation of the relative abundance of the 20 most abundant phyla (Figure 5a) and the 20 most abundant classes (Figure 5b) showed that the microbial composition was similar among the different types of sediments. However, the comparison at the genus level of the 20 most abundant genera revealed some differences between samples (Figure 5c). At the phylum level, the bacteriomes of all the samples (mean relative abundance) were dominated by *Proteobacteria* (45.2%), followed by *Bacteroidota* (or *Bacteroidetes*) (11.9%), *Actinobacteriota* (or *Actinobacteria*) (11.2%), and *Desulfobacterota* (or *Deltaproteobacteria*) (7.3%). On top of that, other less frequent phyla that were present in all the samples were *Campilobacterota* (predominant in sample P2), *Acidobacteriota, Firmicutes, Gemmatimonadota, Myxococcota, Crenarchaeota*, and *Calditrichota*, among others. In terms of class, *Gammaproteobacteria* (27.2%), *Alphaproteobacteria* (18.0%), *Acidimicrobiia* (10.7%), and *Bacteroidia* (9.7%) comprised almost 50% of all the samples. Furthermore, at the genus level, high diversity was found in all the samples. On average, the top 10 genera described in these marine samples were: Unknown *Rhodobacteraceae* (9.0%), *Woeseia* (8.7%), uncultured *Actinomarinales* (8.0%), *Vibrio* (5.8%), *Sulfurovum* (4.7%), *Gammaproteobacteria* B2M28 (2.8%), unknown *Gammaproteobacteria* (2.3%), uncultured *Saprospiraceae* (1.9%), *Desulfosarcinaceae* Sva0081 (1.5%), and uncultured *Syntrophobacterales* (1.4%). Samples CS4 and P2 showed a similar taxa composition to the other samples, but clear differences in abundance, where *Vibrio* and *Sulfurovum* were the dominant genera in each sample, respectively. A test for differential abundance (Table A1) revealed that the phylum *Caldatribacteriota* was significantly more abundant in plastic sediments than in aluminum sediments. At the same time, it showed that when comparing debris sediments to control sediments, *Cyanobacteria* and *Marinimicrobia* were more abundant in can sediments as well as *Campilobacteria, Cloacimonadota*, and *Acetothermia* were significantly more abundant in inner plastic sediments.

### 3.4 Strain collection and identification

**Figure 6:**
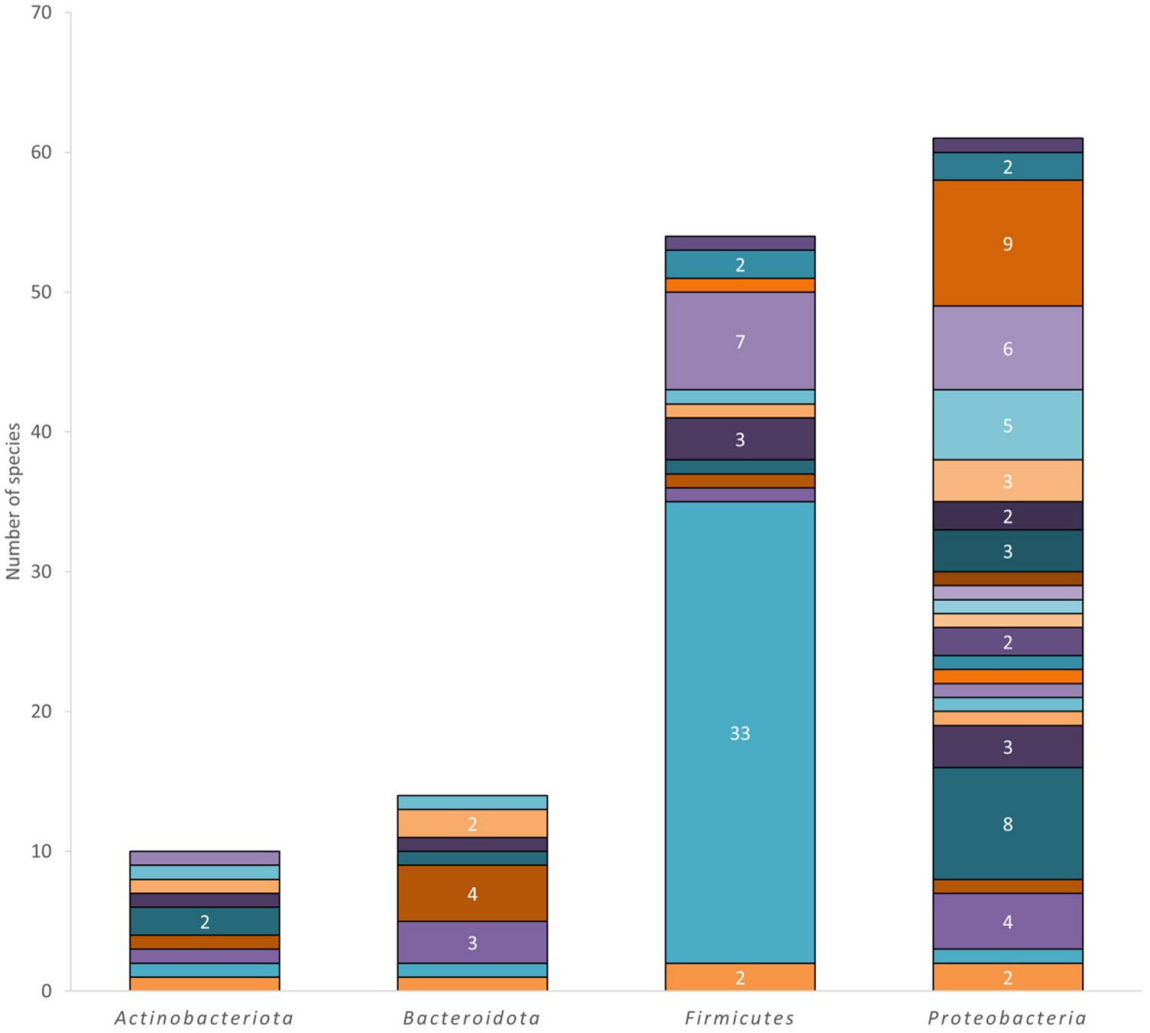
Bar plots showing the distribution into four phyla of the isolated species within the collection. The different colors in each phylum represent one different genus and the numbers indicate the number of isolates identified, which are only written when the number of isolates per genus is greater than two (see Table A2 for detailed information about each strain identified)

Culturing the marine sediments associated with artificial residues yielded a large number of highly diverse microbial colonies, in terms of color and morphology. A total of 170 bacterial strains and one yeast were isolated. All the strains that grew at first under anaerobic conditions showed later the ability to grow in the presence of oxygen. In total, 142 out of 171 strains were identified through colony PCR and 16S and 18S rRNA gene sequencing (Table A2), whereas 29 remained unidentified due to the impossibility to carry out the amplification of these fragments through PCR. The identified bacteria were distributed into four phyla: *Firmicutes, Proteobacteria, Bacteroidota*, and *Actinobacteriota* (Figure 6). *Bacillus* spp. was by far the most abundant genus (33 species identified), followed by *Vibrio* spp. (9), *Erythrobacter* spp. (8), *Planomicrobium* spp (7), *Sulfitobacter* spp. (6) and *Sphingorhabdus* spp. (5) among other genera. Interestingly, the identification of a large fraction of the microorganisms in the collection revealed that some isolates could represent new species, as they held a percentage of identity with the closest type strain below the 98.7% threshold established to circumscribe a new bacterial species (Chun et al., 2018). In particular, isolates M10.2A, M10.9X, and P4.10X with the closest type strains belonging to the genera *Gillisia, Sagittula*, and *Maritalea*, respectively, are potentially new species. Further characterization is needed to determine it.

### 3.5 PET degradation assays

**Figure 7:**
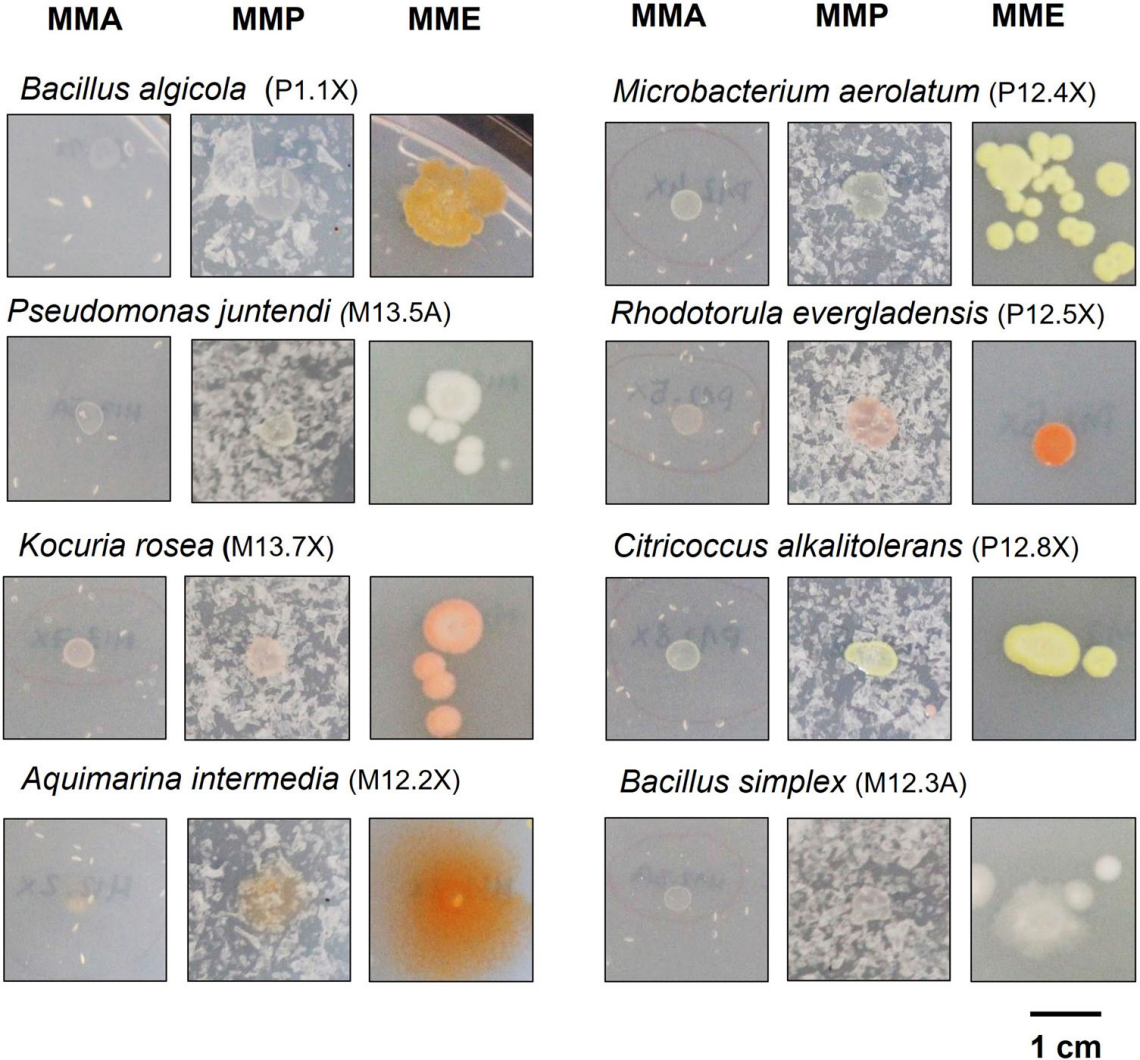
Differential growth of eight selected strains on minimal marine medium (MMA), minimal marine medium supplemented with polyethylene terephthalate (PET) (MMP), and enriched marine medium (MME). MMA was used as a control for the basal growth of the strain without any supplemented carbon source. MMP was used to compare the growth of the isolates in the presence of PET plastic. MME allowed the normal growth of the strain in a rich nutrient marine medium

To test the PET degrading activity of the microbial isolates obtained from marine waste, a preliminary qualitative screening was carried out consisting of a drop assay of bacterial culture in MMA and MMP to check differential growth when PET plastic was present (see Section 2.7). From this preliminary screening, differences in terms of growth after the drop assay performed as described in Section 2 are shown in Figure 7. In the first round of selection, 27 out of the 171 strains tested were selected as they showed increased growth in minimal medium supplemented with PET particles compared to the control medium without PET, after 28 days at 18°C. A second assay with the 27 selected strains was then carried out and led to the further selection of 16 strains with the more obvious differential growth on PET‐containing media. 16S rRNA complete gene sequences were obtained and compared using EzBioCloud thus allowing the identification at the species level (Table A3). A selection of eight of these isolates are shown in Figure 7 and they were identified as members of the species *Bacillus algicola, Pseudomonas juntendi, Kocuria rosea, Aquimarina intermedia, Microbacterium aerolatum, Rhodotorula evergladensis, Citricoccus alkalitolerans*, and *Bacillus simplex*.

**Figure A3:**
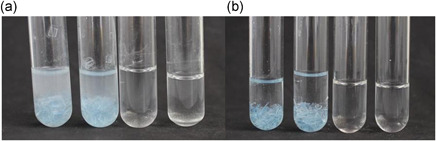
Quantitative assays for polyethylene terephthalate (PET) degradation ability. (a) Negative control tubes at the end of the assay. On the left, the two replicates of the negative control consisting of marine water with PET fragments. A white precipitate of mineral nature appeared after the incubation time, probably due to the change in pH. The two tubes on the right contained only marine water. (b) A representative example of the assay with the isolate M11.3X. All the tubes were inoculated with the bacterium at the beginning of the assay in duplicate, on marine water supplemented with PET particles (left) and marine water without plastic as control (right)

The group of 16 strains selected in the previous assay was incubated for 3 months at 18°C in liquid MMP containing PET particles precisely weighted. The following controls were included in the assay: PET without inoculated bacteria; the medium without neither bacteria nor PET; and each bacterium incubated without plastic. The test resulted in no detectable weight loss of the plastic particles in any sample inoculated with any of the 16 strains. Surprisingly, a small weight loss was detected in the noninoculated controls, in which the liquid became cloudy, appearing a white precipitate (Figure A3). To discard microbial contamination of the controls, the commercial MA medium was inoculated with the cloudy supernatant, which was also observed under the microscope. Both experiments yielded negative results and contamination of the controls was thus discarded. There was one unit decrease in the pH of these control tubes (7.5 ± 0.1) compared with the tubes inoculated with a microorganism, all of which remained at a pH of 8.5 ± 0.3 and exhibited no turbidity in any inoculated tube.

**Figure 8:**
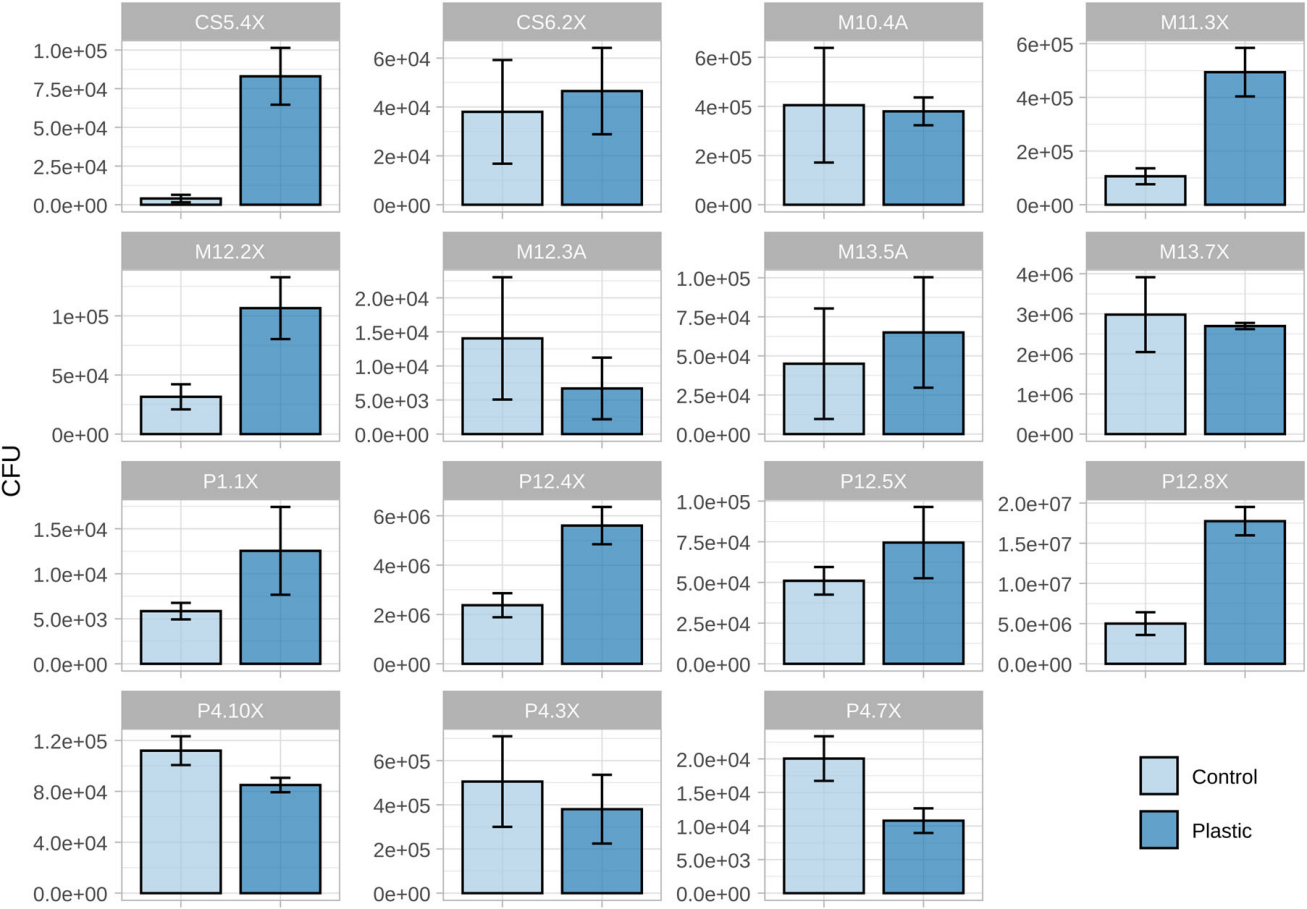
Comparison in colony‐forming units (CFU) count of selected isolates that showed increased growth in polyethylene terephthalate (PET)‐containing medium. Strains were incubated in a PET‐containing medium and control medium without PET at 18°C under shaking (200 rpm) for 3 months. Negative controls yielded no CFU count. Identification of strains based on 16S ribosomal RNA (rRNA) gene sequencing: CS5.4X: *Micrococcus luteus*; CS6.2X: *Aurantimonas coralicida*; M10.4A: *Bacillus zhangzhouensis*; M11.3X: *Idiomarina piscisalsi*; M12.2X: *Aquimarina intermedia*; M12.3A: *Bacillus simplex*; M13.5A: *Pseudomonas juntendi*; M13.7X: *Kocuria rosea*; P1.1X: *Bacillus algicola*; P12.4X: *Microbacterium aerolatum*; P12.5X: *Rhodotorula evergladensis*; P12.8X: *Citricoccus alkalitolerans*; P4.10X: *Maritalea mobilis*; P4.3X: *Meridianimaribacter flavus*; P4.7X: *Microbacterium imperiale*. Accession numbers of deposited 16S rRNA gene sequences and fold‐increase in CFU can be found in Table A3

Substantial differences in bacterial growth were found in the containing‐PET and non‐containing‐PET medium in four of the strains, by comparing cell number (CFU) of the supernatants inoculated in MA medium (Figure 8). The strains that showed an increased growth when PET was present were: *Micrococcus luteus* (CS5.4X, 20.8‐fold increased growth), *Idiomarina piscisalsi* (M11.3X, 4.7‐fold increase), *Citricoccus alkalitolerans* (P12.8X, 3.6‐fold increase), *Aquimarina intermedia* (M12.2X, 3.4‐fold increase), *Microbacterium aerolatum* (P12.4X, 2.4‐fold increase), *Bacillus algicola* (P1.1X, 2.1‐fold increase), and the yeast *Rhodotorula evergladensis* (P12.5X, 1.5‐fold increase) (Table A3).

## 4 DISCUSSION

Artificial residues hold great promise as a source of a huge variety of microorganisms for the bioremediation of plastic waste (Delacuvellerie et al., 2019; Yoshida et al., 2016). The interest in the study of the microbial communities associated with the plastisphere, as well as to other anthropic residues such as glass bottles or ceramic surfaces (McCormick et al., 2014; Oberbeckmann et al., 2016; Pinnell & Turner, 2019), has increased exponentially in the last years. The worldwide problem of plastic contamination in the oceans has led researchers to investigate the impact of these pollutants not only on the surfaces but also in deep‐sea areas (Woodall et al., 2018). Even though these studies shed light on the plastic degradation problem, there are still several questions that need further investigation in this field and more research focusing on other materials such as metal debris would be interesting.

Regarding the bacterial communities inhabiting the marine sediments studied in this study, at the β‐diversity level, the samples analyzed did not cluster together depending on the type of sediment (cans‐inner sediments, plastic‐inner sediments, and control‐external sediments) (Figures 4 and A2). This suggests that the bacterial profile of sediments trapped into artificial residues falls within the diversity of bacterial profiles of similar, natural environments. Interestingly, samples from each type (plastic or metal) displayed similar morphological features under SEM (Figure 2).

The morphology of the microorganisms in the biofilms we studied by SEM is in line with previous descriptions, in which a high diversity of microorganisms, both eukaryotes, and prokaryotes, were found (Bryant et al., 2016; Masó et al., 2016; Reisser et al., 2014). Interestingly, we found numerous fusiform bacteria attached to the plastic surface through fimbriae‐like structures (Figure 2b). Similar shapes have previously been described to inhabit plastic surfaces in marine environments. For example, Bryant et al. (2016), showed a similar microbial community and also reported a bacillary shape that is attached from one pole to the plastic surface. In another study on the plastisphere of microplastics from the Australian shores, the same bacillary shapes with fimbriae‐like structures adhering to the plastic surface were described (Reisser et al., 2014). Furthermore, the well‐known PET degrading bacteria *Ideonella sakaiensis* exhibits attaching appendages when growing on plastic (Figure 2f in Yoshida et al., 2016). Hence, the finding of microorganisms directly attached to the plastic surface points towards the possibility of these bacterial forms being anchored to the plastic substrate to allow its degradation by exoenzymes.

Another interesting morphological trait of the observed microorganisms is the presence of spermatozoid‐shaped bacteria (Figure 2c,e). This bacterial shape may correspond to prosthecate bacteria, particularly the genus *Hyphomonadaceae*, which is abundant in the microbial communities of plastic residues (fig. 5c,f from Bryant et al., 2016; fig. 2 from Zettler et al., 2013) and we have also detected this taxon, although in low abundance, in the marine debris analyzed through high‐throughput 16S rRNA gene sequencing.

In terms of microbiota, our results show that the bacterial profile is very similar between seafloor sediments and internal residue sediments. The microbial composition is characterized by a set of marine bacterial classes (*Gammaproteobacteria, Alphaproteobacteria, Acidimicrobiia*, and *Bacteroidia*) that belong to the phyla *Proteobacteria, Actinobacteriota*, and *Bacteroidota* which have widely been described in surface marine sediments (Hoshino et al., 2020). Indeed, *Gammaproteobacteria* and *Alphaproteobacteria* proved the dominant classes in all the samples analyzed, and they have been reported as the most abundant taxa in samples from pelagic to benthic locations (Petro et al., 2017; Zinger et al., 2011). Moreover, in our study, the phylum *Desulfobacterota* was detected in all the samples. This result correlates with the fact that sulfate concentrations are higher in the surface layers of seafloor sediments (Leloup et al., 2009; Pellerin et al., 2018), which allows the proliferation of species within this phylum, such as members of *Desulfosarcinaceae, Syntrophobacterales, Desulfocapsaceae*, and *Desulfobulbaceae*, all of which were found in the sediments analyzed in this study.

Interestingly, the abundance of the genus *Vibrio* is remarkable in all the samples. Pathogenic bacterial species belonging to *Vibrio* have been widely described in marine environments usually in low abundance and they have also been found in plastic debris (Delacuvellerie et al., 2019; Jacquin et al., 2019; Zettler et al., 2013). *Vibrio* is very resistant to hard conditions and can perform a rapid growth in marine environments in response to an increase of nutrients (Westrich et al., 2018). Another interesting fact is that PET bottle P2 was dominated by *Sulfurovum* while this genus remained in low abundance in the other samples. Species from the genus *Sulfurovum* are chemolithoautotrophic sulfur‐oxidizing bacteria that are primary producers in marine sediments communities (Mori et al., 2018) and even have been described to be the dominant taxon in seafloor sediments in some localizations (Sun et al., 2020).

The microbial composition we have found is similar to that reported in a variety of studies carried out on the biofilm that directly colonizes the plastic surface (Amaral‐Zettler et al., 2020; Delacuvellerie et al., 2019; Oberbeckmann et al., 2016). A recent review on colonization and plastic biodegradation in the marine environment (Jacquin et al., 2019), summarizes that the surface of plastic residues are generally quickly colonized by *Gammaproteobacteria* and *Alphaproteobacteria*, and then, with time, *Bacteroidota* also becomes an important group in the biofilm.

The microbial profiles observed in the collection of culturable strains we set are in accordance with the previous results reported by several authors. This collection of 171 microbial isolates includes strains of 53 different genera distributed among the phyla *Firmicutes, Proteobacteria, Bacteroidota*, and *Actinobacteriota*. Specifically, *Proteobacteria*, which is one of the most common phyla in most of the biomes, is also the most abundant phylum associated with plastic residues worldwide (Roager & Sonnenschein, 2019). Among the recurrent alphaproteobacterial families found in such environments are *Erythrobacteraceae* and *Rhodobacteraceae*, which in our collection are represented by the eight genera: two belonging to *Erythrobacteraceae* (*Altererythrobacter, Erythrobacter*) and six belonging to *Rhodobacteraceae* (*Epibacterium, Maliponia, Ruegeria, Sagittula, Sulfitobacter*, and *Yoonia*). Moreover, the eight representative genera of the phylum *Bacteroidota* belonged to the *Flavobacteriaceae* family, which is, again, a common plastic debris‐associated taxa (Amaral‐Zettler et al., 2020; Jacquin et al., 2019). The abundance of *Firmicutes* is linked to the high number of *Bacillus* spp, (33 species isolated in total) we found. This genus has been reported as a marine plastic colonizer and degrader (Delacuvellerie et al., 2019; Oberbeckmann et al., 2015; Ribitsch et al., 2011).

The diversity of microorganisms found on artificial debris, the presence of biofilms and plastic adhesion fimbriae‐like structures, and the taxonomic identity of some of the taxa suggest a possible role in plastic biodegradation of some of the bacteria of the collection we set and characterized. The quantitative PET degradation assay with the selected strains yielded no significant loss of non‐pretreated PET particles weight. However, this is not particularly surprising givien the fact that PET is very resistant to biodegradation due to its compact structure, hence heat or oxidative pretreatments are usually needed to enhance biodegradation (Gewert et al., 2015). Nevertheless, we observed an increased growth (measured as CFU count variation), of seven of the isolates when PET was present as the sole carbon source in the medium, suggesting the capability of some strains to degrade plastic or plastic additives, such as plasticizers, antioxidants, light and heat stabilizers, pigments or slip reagents that are usually added to plastics to enhance their structural properties. These compounds are commonly not covalently bonded to the plastic polymer; therefore, they can more easily leak out from the plastic structure to the liquid phase (Hahladakis et al., 2018). Remarkably, the strain of *Micrococcus luteus* we tested, showed a 20‐fold increase in CFUs when the minimal medium was supplemented with PET particles compared to a non‐supplemented‐PET medium. This is not the first time that *Micrococcus luteus* has been described to potentially degrade plastic (Montazer et al., 2018; Sivasankari & Vinotha, 2014), and its degrading ability seems to be associated with its ability to form biofilm in plastic surfaces (Blakeman et al., 2019; Feng et al., 2011). The isolates identified as *Idiomarina piscisalsi, Citricoccus alkalitolerans, Aquimarina intermedia*, and *Microbacterium aerolatum* which showed roughly a two‐ to four‐fold increase in growth in PET, have been sparsely studied in previous works regarding plastic‐degrading activity. Specifically, *Idiomarina* has been recently reported to possibly assist in the formation of biofilms on the surface of PET particles, although it showed no significant PET degradation (Gao & Sun, 2021). On the contrary, although there is no previous report on the ability of *Bacillus algicola* (which showed double CFU count when incubated with PET) to degrade plastic polymers, other species and strains within the genus have been described as degraders of polystyrene, polypropylene, polyethylene, and PET microplastic particles (Auta et al., 2017; Wright, Bosch, et al., 2021) as well as polyvinyl chloride (Giacomucci et al., 2019). Finally, the yeast *Rhodotorula evergladensis*, which showed a tiny increase in growth on PET in our study, has been previously reported to degrade plasticizers (Gartshore et al., 2003).

Taken together, our results suggest that the marine waste‐associated microbiota hold potential as a source of biotechnological interesting strains for plastic or plastic‐related compounds.

## CONFLICT OF INTERESTS

None declared.

## ETHICS STATEMENT

None required.

## AUTHOR CONTRIBUTIONS

**Àngela Vidal‐Verdú**: Conceptualization (equal); Data curation (lead); Investigation (lead); Writing – original draft (lead); Writing – review and editing (equal); **Adriel Latorre‐Pérez**: Conceptualization (supporting); Formal analysis (lead); Writing – review and editing (equal); **Esther Molina‐Menor**: Investigation (supporting); Writing – original draft (supporting); Writing – review and editing (equal); **Joaquin Baixeras**: Investigation (Supporting); Supervision (Supporting); Writing – review and editing (equal); **Juli Peretó**: Conceptualization (equal); Writing – review and editing (equal); **Manuel Porcar**: Conceptualization (equal); Writing – review and editing (equal).

## Data Availability

The datasets generated for this study can be found in online repositories. Raw reads are available in the NCBI repository (BioProject accession PRJNA704512: https://www.ncbi.nlm.nih.gov/bioproject/PRJNA704512). 16S and 18S rRNA gene sequences are available in GenBank under accession numbers MZ437807‐MZ437945, MZ604909‐MZ604910, MZ604692, MZ994595‐MZ994596, and MW785249. The code and the results of the bioinformatics and statistical analyses (including taxonomy tables of absolute and relative abundances and the BIOM table) have been uploaded to GitHub (https://github.com/adlape95/Living-in-a-bottle).

## Appendix

**Table A1:** Results of comparing plastic inner sediments, aluminum cans inner sediments, and control sediments from the seabed by DESeq. 2 test

***Phylum level***											
**Base mean abundance**			**log2FoldChange**	**lfcSE**			***p* value**		**FDR‐adjusted *p* value**	**Domain**	**Phylum**
**Can sediment versus control sediment**											
**Reference: Control sediment**											
**Test: DESeq. 2**											
221.353			2.046	0.610			0.001		2.89E−02	d__Bacteria	p__Cyanobacteria
128.105			1.612	0.463			0.000		2.89E−02	d__Bacteria	p__Marinimicrobia_(SAR406_clade)
**Plastic sediment versus control sediment**											
**Reference: Control sediment**											
**Test: DESeq. 2**											
9090.508		5.066			1.36		1.97E−04		7.39E−03	d__Bacteria	p__Campilobacterota
69.709		5.242			1.57		8.29E−04		2.07E−02	d__Bacteria	p__Cloacimonadota
68.653		22.730			3.68		6.46E−10		4.84E−08	d__Bacteria	p__Acetothermia
**Plastic sediment versus can sediment**											
**Reference: Can sediment**											
**Test: DESeq. 2**											
24.250			21.853		2.916		6.74E−14		5.12E−12	d__Bacteria	p__Caldatribacteriota
***Genus level***											
**Base mean abundance**	**Log2FoldChange**				**lfcSE**	***p* value**		**FDR‐adjusted *p* value**	**Phylum**	**Family**	**Genus**
**Can sediment versus control sediment**											
**Reference: Control sediment**											
**Test: DESeq. 2**											
172.967	23.916				3.678	7.93E−11		8.07E−09	p__Firmicutes	f__Lachnospiraceae	g__Shuttleworthia
157.511	23.795				3.051	6.29E−15		3.20E−12	p__Firmicutes	f__Hungateiclostridiaceae	g__Fastidiosipila
33.656	−23.170				3.381	7.20E−12		9.16E−10	p__Firmicutes	f__Clostridia_UCG‐014	g__Clostridia_UCG‐014
129.622	2.701				0.690	9.02E−05		5.10E−03	p__Bacteroidota	f__Lentimicrobiaceae	g__Lentimicrobiaceae
103.293	3.529				0.936	1.64E−04		8.36E−03	p__Bacteroidota	f__Prolixibacteraceae	g__uncultured
63.609	22.529				3.086	2.89E−13		4.90E−11	p__Firmicutes	f__Hungateiclostridiaceae	g__Mageibacillus
10.991	20.088				3.682	4.87E‐08		3.54E−06	p__Firmicutes	f__Veillonellaceae	g__Veillonella
55.211	22.335				3.036	1.89E−13		4.81E−11	p__Firmicutes	f__Streptococcaceae	g__Lactococcus
21.466	7.658				2.282	7.91E−04		3.35E−02	p__Proteobacteria	f__Rhodobacteraceae	g__Confluentimicrobium
23.666	−2.979				0.855	4.94E−04		2.29E−02	p__Proteobacteria	f__Kangiellaceae	g__Kangiella
21.844	21.042				3.680	1.08E−08		9.14E−07	p__Firmicutes	f__Lachnospiraceae	g__[Ruminococcus]_torques_group
10.304	20.000				3.682	5.58E_08		3.55E−06	p__Proteobacteria	f__Rhodobacteraceae	g__Pseudoruegeria
**Plastic sediment versus control sediment**											
**Reference: Control sediment**											
**Test: DESeq. 2**											
454.017	10.661				3.196	8.51E−04		2.66E−02	p__Actinobacteriota	f__Propionibacteriaceae	g__Cutibacterium
18,607.676	6.694				2.024	9.41E−04		2.82E−02	p__Campilobacterota	f__Sulfurovaceae	g__Sulfurovum
672.863	9.176				2.510	2.57E−04		8.41E−03	p__Bacteroidota	f__Chlorobiaceae	g__Prosthecochloris
241.181	6.801				2.100	1.20E−03		3.46E−02	p__Chloroflexi	f__661239	g__661239
1402.831	9.537				3.065	1.86E−03		4.79E−02	p__Chloroflexi	f__GIF3	g__GIF3
41.074	−23.332				3.632	1.32E−10		4.75E−08	p__Firmicutes	f__Erysipelotrichaceae	g__Dubosiella
136.694	6.841				2.179	1.69E−03		4.51E−02	p__Cloacimonadota	f__MSBL8	g__MSBL8
52.605	22.421				3.539	2.35E−10		5.65E−08	p__Bacteroidota	f__SJA‐28	g__SJA‐28
44.815	22.197				3.679	1.61E−09		1.29E−07	p__Actinobacteriota	f__Micrococcaceae	g__Rothia
15.691	−22.020				3.633	1.35E−09		1.22E−07	p__Bacteroidota	f__Rikenellaceae	g__Alistipes
12.107	20.379				3.682	3.12E−08		1.25E−06	p__Halobacterota	f__Methanoregulaceae	g__Methanolinea
12.491	20.419				3.682	2.93E−08		1.24E−06	p__Proteobacteria	f__Rhizobiaceae	g__Lentilitoribacter
143.561	23.346				3.599	8.74E−11		4.75E−08	p__Acetothermia	f__Acetothermiia	g__Acetothermiia
23.229	−22.563				3.632	5.24E−10		9.43E−08	p__Bacteroidota	f__Muribaculaceae	g__Muribaculaceae
30.963	21.683				3.680	3.80E−09		2.11E−07	p__Proteobacteria	f__Pasteurellaceae	g__Rodentibacter
30.963	21.683				3.680	3.80E−09		2.11E−07	p__Actinobacteriota	f__Demequinaceae	g__Demequina
9.854	20.094				3.683	4.87E−08		1.67E−06	p__Proteobacteria	f__Rhodobacteraceae	g__Tropicibacter
51.876	22.402				3.679	1.14E−09		1.22E−07	p__Chloroflexi	f__FS117‐23B‐02	g__FS117‐23B‐02
60.568	22.616				3.679	7.87E−10		1.13E−07	p__Chloroflexi	f__FW22	g__FW22
28.247	21.555				3.680	4.70E−09		2.42E−07	p__Chloroflexi	f__uncultured	g__uncultured
11.692	20.331				3.682	3.36E−08		1.27E−06	p__Proteobacteria	f__Rhodobacteraceae	g__Pseudophaeobacter
39.111	22.008				3.679	2.21E−09		1.57E−07	p__Chloroflexi	f__AB‐539‐J10	g__AB‐539‐J10
49.975	22.348				3.679	1.24E−9		1.22E‐07	p__Chloroflexi	f__Dehalococcoidia	g__Dehalococcoidia
18.741	20.981				3.681	1.20E−08		5.38E−07	p__Aenigmarchaeota	f__Aenigmarchaeales	NA
12.303	7.012				2.206	1.48E−03		4.11E−02	p__Acidobacteriota	f__Acanthopleuribacteraceae	g__Acanthopleuribacter
37.753	21.959				3.679	2.40E−09		1.57E−07	p__Chloroflexi	f__GIF9	g__GIF9
10.548	20.188				3.683	4.21E−08		1.51E−06	p__Proteobacteria	f__Caulobacteraceae	g__Brevundimonas
23.630	21.306				3.680	7.06E−09		3.39E−07	p__Chloroflexi	f__Sh765B‐AG‐111	g__Sh765B‐AG‐111
**Plastic sediment versus can sediment**											
**Reference: Can sediment**											
**Test: DESeq. 2**											
420.185	8.385				2.380	4.26E−04		1.21E−02	p__Actinobacteriota	f__Propionibacteriaceae	g__Cutibacterium
617.280	10.016				2.614	1.27E−04		3.81E−03	p__Bacteroidota	f__Chlorobiaceae	g__Prosthecochloris
125.086	−24.614				3.186	1.11E−14		5.99E−12	p__Firmicutes	f__Hungateiclostridiaceae	g__Fastidiosipila
179.331	−23.932				3.214	9.69E−14		1.74E−11	p__Firmicutes	f__Veillonellaceae	g__Megasphaera
112.919	24.056				3.384	1.17E−12		1.26E−10	p__Proteobacteria	f__Sedimenticolaceae	g__Candidatus_Thiodiazotropha
56.910	−23.544				3.157	8.78E−14		1.74E−11	p__Firmicutes	f__Erysipelotrichaceae	g__Dubosiella
48.636	−23.324				3.199	3.10E−13		4.18E−11	p__Firmicutes	f__Hungateiclostridiaceae	g__Mageibacillus
9.616	−21.118				3.387	4.51E−10		1.74E−08	p__Firmicutes	f__Veillonellaceae	g__Veillonella
56.335	23.106				3.384	8.64E−12		7.78E−10	p__Caldatribacteriota	f__JS1	g__JS1
19.112	−22.040				3.385	7.47E−11		3.36E−09	p__Firmicutes	f__Lachnospiraceae	g__[Ruminococcus]_torques_group
41.129	22.668				3.385	2.12E−11		1.27E−09	p__Actinobacteriota	f__Micrococcaceae	g__Rothia
10.782	20.807				3.388	8.16E−10		2.75E−08	p__Halobacterota	f__Methanoregulaceae	g__Methanolinea
8.932	−21.035				3.387	5.29E−10		1.90E−08	p__Proteobacteria	f__Magnetospiraceae	g__Magnetovibrio
35.895	22.475				3.385	3.13E−11		1.69E−09	p__Chloroflexi	f__AB‐539‐J10	g__AB‐539‐J10
45.865	22.821				3.384	1.55E−11		1.05E−09	p__Chloroflexi	f__Dehalococcoidia	g__Dehalococcoidia
22.443	21.818				3.222	1.28E−11		9.89E−10	p__Asgardarchaeota	f__Lokiarchaeia	g__Lokiarchaeia
34.648	22.428				3.385	3.44E−11		1.69E−09	p__Chloroflexi	f__GIF9	g__GIF9
11.110	−6.714				1.654	4.90E−05		156E−03	p__Verrucomicrobiota	f__Rubritaleaceae	g__Rubritalea
21.686	21.623				3.386	1.69E−10		7.03E−09	p__Chloroflexi	f__Sh765B‐AG‐111	g__Sh765B‐AG‐111

*Note*: Only significant results (FDR‐adjusted *p* < 0.05) are shown. Positive log2FoldChange values mean that the taxon is underrepresented in the reference, while negative values mean that the taxon is overrepresented in the reference.

**Table A2:** List of the strains identified in the collection, with the closest type strain, accession number, ID percentage, and the GenBank accession number for the 16S or 18S rRNA gene sequences obtained in this study

Sample	Closest type strain	Accession number	ID %	GenBank accession number
P1.1A	*Marinobacter similis*	CP007151	99.58	MZ437807
P1.2A	*Aurantimonas coralicida*	ATXK01000033	100	MZ437808
P1.3A	*Sulfitobacter sabulilitoris*	MK726099	98.71	MZ437809
P1.4A	*Bacillus beringensis*	FJ889576	99.1	MZ437810
P2.1A	*Bacillus endophyticus*	AF295302	99.67	MZ437811
P2.2A	*Bacillus oryzaecorticis*	KF548480	98.67	MZ437812
P2.3A	*Bacillus altitudinis*	ASJC01000029	99.58	MZ437813
P2.4A	*Pontibacillus salipaludis*	LN872943	99.73	MZ437814
P2.5A	*Bacillus firmus*	BCUY01000205	98.6	MZ437815
P3.1A	*Erythrobacter arachoides*	KU302715	98.36	MZ437816
P3.2A	*Bacillus Altitudinis*	ASJC01000029	99.86	MZ437817
P3.3A	*Planomicrobium alkanoclasticum*	AF029364	99.58	MZ437818
P3.4A	*Bacillus drentensis*	AJ542506	99.06	MZ437819
P3.5A	*Erythrobacter longus*	JMIW01000006	98.61	MZ437820
P4.1A	*Bacillus wiedmannii*	LOBC01000053	99.6	MZ437821
P4.2A	*Jiella aquimaris*	KJ620984	100	MZ437822
P4.3A	*Aeromicrobium alkaliterrae*	AY822044	97.57	MZ437823
P4.4A	*Aurantimonas coralicida*	ATXK01000033	99.54	MZ437824
P4.5A	*Erythrobacter insulae*	MK991775	98.91	MZ437825
M10.1A	*Kocuria palustris*	Y16263	100	MZ437826
M10.2A	*Gillisia mitskevichiae*	jgi.1107713	97.57	MZ994595
M10.3A	*Sagittula stellata*	AAYA01000003	97.81	MZ437828
M10.4A	*Bacillus zhangzhouensis*	JOTP01000061	99.60	MZ437829
M10.5A	*Gramella sediminilitoris*	KU696541	98.53	MZ437830
M11.1A	*Planomicrobium alkanoclasticum*	AF029364	99.85	MZ437831
M11.2A	*Sulfitobacter undariae*	KM275624	98.42	MZ437832
M11.3A	*Hoeflea halophila*	OCPC01000011	99.71	MZ437833
M11.4A	*Pontixanthobacter luteolus*	AY739662	99.61	MZ437834
M11.5A	*Sulfitobacter mediterraneus*	JASH01000023	98.48	MZ437835
M12.1A	*Pseudomonas juntendi*	MK680061	99.61	MZ437836
M12.3A	*Bacillus simplex*	BCVO01000086	100	MZ437837
M12.4A	*Hoeflea halophila*	OCPC01000011	99.67	MZ437838
M12.5A	*Halobacillus locisalis*	AY190534	99.83	MZ437839
M13.1A	*Bacillus toyonensis*	CP006863	99.87	MZ437840
M13.3A	*Sulfitobacter undariae*	KM275624	97.99	MZ437841
M13.4A	*Bhargavaea beijingensis*	EF371374	99.33	MZ437842
M13.5A	*Pseudomonas juntendi*	MK680061	99.87	MZ437843
CS5.1A	*Sediminicola arcticus*	KM576847	97.43	MZ437844
CS5.2A	*Sediminicola arcticus*	KM576847	98.29	MZ437845
CS5.3A	*Planomicrobium alkanoclasticum*	AF029364	99.36	MZ437846
CS5.4A	*Pseudidiomarina aquimaris*	PIPT01000016	98.98	MZ437847
CS5.5A	*Erythrobacter citreus*	AF118020	98.49	MZ437848
CS6.1A	*Hoeflea halophila*	OCPC01000011	98.98	MZ437849
CS6.2A	*Aurantimonas coralicida*	ATXK01000033	100.00	MZ437850
CS6.4A	*Bacillus aciditolerans*	MG589508	99.49	MZ437851
CS6.5A	*Gramella salexigens*	CP018153	98.67	MZ437852
P12.1A	*Altererythrobacter luteolus*	AY739662	99.54	MZ437853
P12.3A	*Bacillus aciditolerans*	MG589508	99.45	MZ437854
P12.4A	*Bacillus zhangzhouensis*	JOTP01000061	99.84	MZ437855
P12.5A	*Bacillus megaterium*	JJMH01000057	99.78	MZ437856
P12.6A	*Bacillus filamentosus*	KF265351	99.54	MZ437857
P1.1X	*Bacillus algicola*	NR_117184.1	100	MZ604909
P1.2X	*Erythrobacter arachoides*	KU302715	98.61	MZ437858
P1.4X	*Terribacillus saccharophilus*	AB243845	99.59	MZ437859
P1.5X	*Sphingorhabdus flavimaris*	AY554010	99.58	MZ437860
P1.7X	*Halobacillus locisalis*	AY190534	100	MZ437861
P1.8X	*Sulfitobacter mediterraneus*	JASH01000023	97.99	MZ437862
P2.1X	*Bacillus oceanisediminis*	GQ292772	99.14	MZ437863
P2.2X	*Fictibacillus halophilus*	KP265300	99.7	MZ437864
P2.3X	*Bacillus firmus*	BCUY01000205	98.61	MZ437865
P2.4X	*Cytobacillus firmus*	BCUY01000205	98.95	MZ437866
P2.5X	*Alkalihalobacillus algicola*	AY228462	99.13	MZ437867
P2.6X	*Brevibacterium frigoritolerans*	AM747813	100	MZ437868
P2.7X	*Solibacillus isronensis*	AMCK01000046	99.86	MZ437869
P3.2X	*Bacillus algicola*	AY228462	98.19	MZ437870
P3.4X	*Pseudomonas juntendi*	MK680061	99.82	MZ437871
P3.5X	*Bacillus altitudinis*	ASJC01000029	99.75	MZ437872
P3.7X	*Actibacter haliotis*	KC193210	98.83	MZ437873
P3.8X	*Sphingorhabdus flavimaris*	AY554010	99.31	MZ437874
P4.1X	*Solibacillus isronensis*	AMCK01000046	100	MZ437875
P4.3X	*Meridianimaribacter flavus*	jgi.1076156	99.71	MZ437876
P4.5X	*Rhizobium marinum*	JMQK01000051	97.87	MZ437877
P4.7X	*Microbacterium imperiale*	X77442	100.00	MZ437878
P4.8X	*Rhizobium marinum*	JMQK01000051	100.00	MZ437879
P4.9X	*Nocardioides nitrophenolicus*	AF005024	98.72	MZ437880
P4.10X	*Maritalea myrionectae*	AUHV01000006	98.25	MZ994596
M10.1X	*Bacillus altitudinis*	ASJC01000029	99.61	MZ437882
M10.2X	*Gramella sediminilitoris*	KU696541	98.68	MZ437883
M10.4X	*Planomicrobium alkanoclasticum*	AF029364	100	MZ437884
M10.5X	*Piscibacillus halophilus*	FM864227	98.78	MZ437885
M10.6X	*Planomicrobium alkanoclasticum*	AF029364	99.61	MZ437886
M10.7X	*Sulfitobacter sabulilitois*	MK726099	98.14	MZ437887
M10.8X	*Gillisia hiemivivida*	AY694006	97.45	MZ437888
M10.9X	*Sagittula stellata*	AAYA01000003	97.81	MW785249
M11.1X	*Bacillus endophyticus*	AF295302	100	MZ437890
M11.2X	*Ruegeria atlantica*	CYPU01000053	99.33	MZ437891
M11.3X	*Idiomarina piscisalsi*	AB619724	99.36	MZ437892
M11.4X	*Bacillus pseudomycoides*	ACMX01000133	100.00	MZ437893
M11.5X	*Bacillus altitudinis*	ASJC01000029	99.47	MZ437894
M11.6X	*Erythrobacter arachoides*	KU302715	99.16	MZ437895
M11.7X	*Bacillus horikoshii*	X76443	98.74	MZ437896
M11.8X	*Sphingorhabdus flavimaris*	AY554010	99.58	MZ437897
M11.9X	*Altererythrobacter luteolus*	AY739662	99.57	MZ437898
M11.10X	*Planomicrobium alkanoclasticum*	AF029364	99.6	MZ437899
M11.11X	*Aquamicrobium lusatiense*	AJ132378	90.26	MZ437900
M11.12X	*Bacillus altitudinis*	ASJC01000029	100.00	MZ437901
M12.1X	*Erythrobacter arachoides*	KU302715	98.64	MZ437902
M12.2X	*Aquimarina intermedia*	jgi.1107908	99.71	MZ437903
M12.3X	*Lutimonas vermicola*	EF108218	99.28	MZ437904
M12.4X	*Parasphingorhabdus flavimaris*	AY554010	99.71	MZ437905
M13.1X	*Bacillus altitudinis*	ASJC01000029	100.00	MZ437906
M13.2X	*Halobacillus litoralis*	X94558	99.86	MZ437907
M13.3X	*Microbulbifer echini*	KJ789957	99.76	MZ437908
M13.4X	*Alkalihalobacillus algicola*	AY228462	99.85	MZ437909
M13.7X	*Kocuria salina*	LT674162	99.31	MZ437910
CS4.1X	*Yoonia litorea*	jgi.1096519	99.74	MZ437911
CS4.3X	*Vibrio comitans*	DQ922915	99.45	MZ437912
CS4.4X	*Mesobacillus subterraneus*	RSFW01000004	99.52	MZ437913
CS4.5X	*Agromyces indicus*	HM036655	98.81	MZ437914
CS5.2X	*Bacillus horikoshii*	X76443	99.07	MZ437915
CS5.3X	*Planomicrobium alkanoclasticum*	AF029364	99.4	MZ437916
CS5.4X	*Micrococcus luteus*	CP001628	99.82	MZ437917
CS6.1X	*Sphingorhabdus flavimaris*	AY554010	99.32	MZ437918
CS6.2X	*Aurantimonas coralicida*	ATXK01000033	100.00	MZ437919
CS6.3X	*Gramella salexigens*	CP018153	98.87	MZ437920
CS6.4X	*Microbulbifer echini*	KJ789957	99.2	MZ437921
CS6.5X	*Sphingorhabdus flavimaris*	AY554010	99.31	MZ437922
CS6.6X	*Gillisia mitskevichiae*	jgi.1107713	99.74	MZ437923
CS6.7X	*Erythrobacter citreus*	AF118020	99.04	MZ437924
P12.4X	*Microbacterium aerolatum*	BJUW01000027	99.76	MZ604910
P12.5X	*Rhodotorula mucilaginosa*	KU167832.1	100.00	MZ604692
P12.6X	*Yoonia rosea*	jgi.1085777	99.06	MZ437925
P12.7X	*Ruegeria arenilitoris*	FXYG01000008	99.6	MZ437926
P12.8X	*Citricoccus alkalitolerans*	AY376164	99.21	MZ437927
P12.9X	*Bacillus maritimus*	KP317497	98.04	MZ437928
P12.10X	*Tessaracoccus rhinocerotis*	KT215777	97.89	MZ437929
P12.11X	*Bacillus beringensis*	FJ889576	98.98	MZ437930
P12.12X	*Bacillus altitudinis*	ASJC01000029	99.58	MZ437931
P12.13X	*Bacillus firmus*	BCUY01000205	97.85	MZ437932
P12.14X	*Bacillus aryabhattai*	EF114313	100.00	MZ437933
P12.15X	*Ruegeria arenilitoris*	FXYG01000008	100.00	MZ437934
P1.1D	*Pseudoalteromonas piscicida*	CP011925	100.00	MZ437935
P1.2D	*Vibrio hyugaensis*	LC004912	99.86	MZ437936
P1.5D	*Vibrio azureus*	LC004912	99.59	MZ437937
P6.1D	*Vibrio alginolyticus*	CP006718	99.34	MZ437938
P6.2D	*Vibrio hyugaensis*	LC004912	99.55	MZ437939
M10.3D	*Epibacterium mobile*	jgi.1108012	100	MZ437940
M10.5D	*Vibrio azureus*	BATL01000140	100.00	MZ437941
M10.6D	*Vibrio shilonii*	ABCH01000080	99.03	MZ437942
M10.7D	*Vibrio hyugaensis*	LC004912	99.86	MZ437943
M10.10D	*Vibrio hyugaensis*	LC004912	99.85	MZ437944
M10.11D	*Tenacibaculum mesophilum*	jgi.1107970	99.87	MZ437945

*Note*: The identification code of the strains corresponds to the sediments from which it was isolated (CS, control sediments; M, can inside‐sediments; P, plastic inside‐sediments; and a number).

**Table A3:** List of selected isolates that showed enhanced growth in PET‐containing medium, with the closest type strain, GenBank accession number for the 16S and 18 rRNA gene sequences, and results obtained in the quantitative assay

Isolate	Closest Type Strain	Quantitative assay (CFU in minimal marine medium supplemented with PET/CFU in minimal marine medium)	GenBank accession number
P1.1X	*Bacillus algicola*	2.1	MZ604909
P4.3X	*Meridianimaribacter flavus*	0.8	MZ437876
P4.7X	*Microbacterium imperiale*	0.5	MZ437878
M13.5A	*Pseudomonas juntendi*	1.4	MZ437843
M13.7X	*Kocuria rosea*	0.9	MZ437910
M11.3X	*Idiomarina piscisalsi*	4.7	MZ437892
P4.10X	*Maritalea mobilis*	0.8	MZ437881
P12.10X	*Tessaracoccus rhinocerotis*	*	MZ437929
CS5.4X	*Micrococcus luteus*	20.8	MZ437917
CS6.2X	*Aurantimonas coralicida*	1.2	MZ437919
M12.2X	*Aquimarina intermedia*	3.4	MZ437903
P12.4X	*Microbacterium aerolatum*	2.4	MZ604910
P12.5X	*Rhodotorula evergladensis*	1.5	MZ604692
P12.8X	*Citricoccus alkalitolerans*	3.6	MZ437927
M10.4A	*Bacillus zhangzhouensis*	0.9	MZ437829
M12.3A	*Bacillus simplex*	0.5	MZ437837

Abbreviations: CFU, colony‐forming units; PET, polyethylene terephthalate.

**Tessaracoccus rhinocerotis* yielded an uncountable number of colonies; therefore, its differential growth was not measured.
